# The specificity of neuroprotection by antioxidants

**DOI:** 10.1186/1423-0127-16-98

**Published:** 2009-11-05

**Authors:** Yuanbin Liu, David R Schubert

**Affiliations:** 1 Cellular Neurobiology Laboratory, The Salk Institute for Biological Studies,10010 N. Torrey Pines Road, La Jolla, California 92037-1099 USA

## Abstract

**Background:**

Reactive oxygen species (ROS) play an important role in aging and age-related diseases such as Parkinson's disease and Alzheimer's disease. Much of the ROS production under conditions of toxic stress is from mitochondria, and multiple antioxidants prevent ROS accumulation. The aim of this study is to examine the specificity of the interaction between the antioxidants and ROS production in stressed cells.

**Methods:**

Using fluorescent dyes for ROS detection and mitochondrial inhibitors of known specificities, we studied ROS production under three conditions where ROS are produced by mitochondria: oxidative glutamate toxicity, state IV respiration induced by oligomycin, and tumor necrosis factor-induced cell death.

**Results:**

We demonstrated that there are at least four mitochondrial ROS-generating sites in cells, including the flavin mononucleotide (FMN) group of complex I and the three ubiquinone-binding sites in complexes I, II and III. ROS production from these sites is modulated in an insult-specific manner and the sites are differentially accessible to common antioxidants.

**Conclusion:**

The inhibition of ROS accumulation by different antioxidants is specific to the site of ROS generation as well as the antioxidant. This information should be useful for devising new interventions to delay aging or treat ROS-related diseases.

## Background

The production of reactive oxygen species (ROS) is greatly increased under many conditions of toxic stress [1,2]. However, existing antioxidants appear to be relatively ineffective in combating these problems, either because they cannot reach the site of ROS production, which is frequently within mitochondria, or because of their poor ability to scavenge the damaging ROS. Identifying compounds that directly block mitochondrial ROS production may be a novel way to inhibit oxidative stress, and perhaps delay aging and treat mitochondrial ROS-related diseases. However, it remains a challenge to define both the normal and pathologically relevant sites of ROS formation in the mitochondrial electron transport chain (ETC) and to find clinically useful agents that can minimize mitochondrial ROS production.

**Figure 1:**
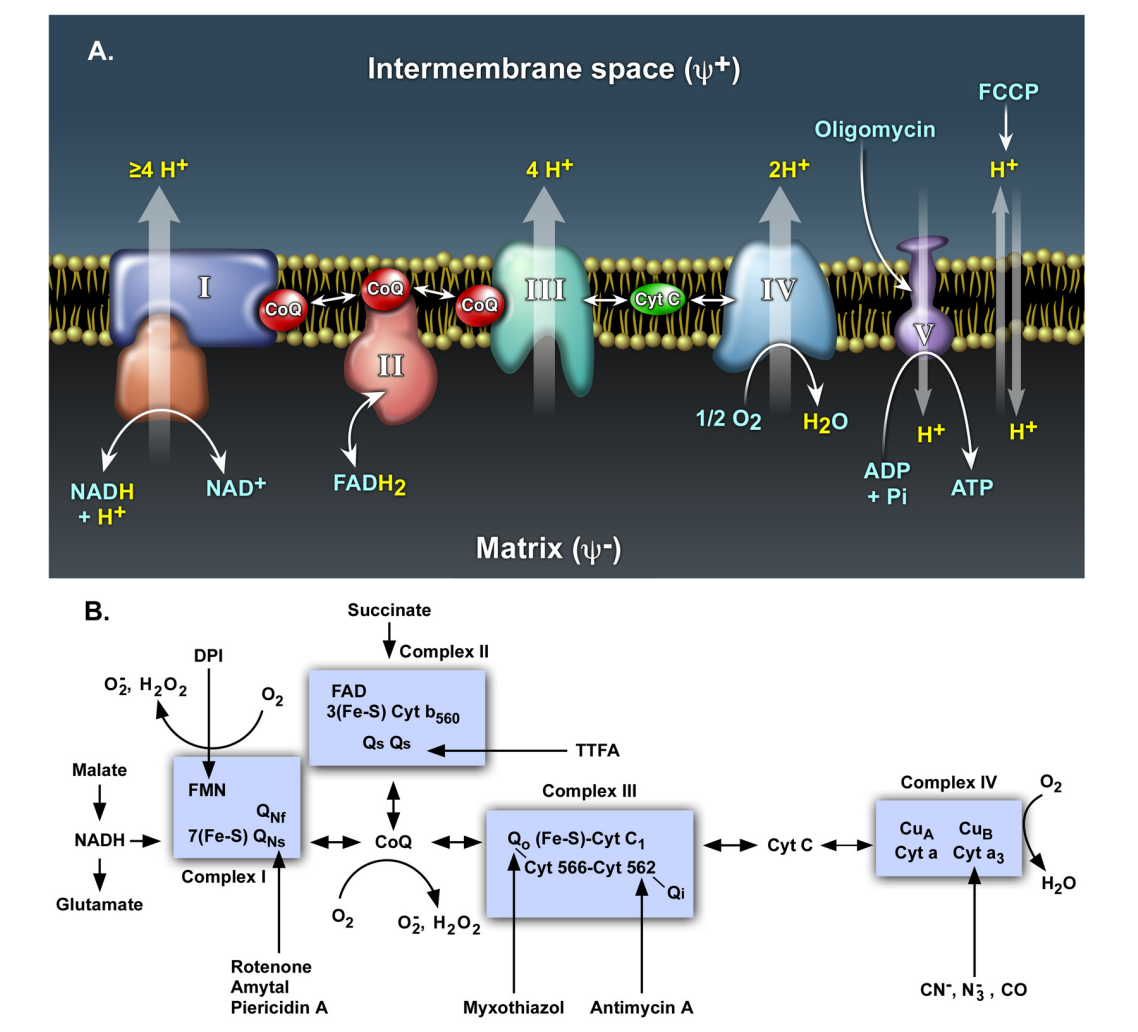
**Oxidative Phosphorylation and the Mitochondrial Electron Transport Chain**. *A*: Oxidative phosphorylation: the membrane topology of mitochondrial complexes, the sites of proton translocation and the targets of agents that affect the transmembrane proton gradient. *B*: The mitochondrial electron transport chain: the sites of ROS generation and the sites of action of commonly used respiratory inhibitors.

The mitochondrial ETC is composed of a series of electron carriers (flavoproteins, iron-sulfur proteins, ubiquinone and cytochromes) that are arranged spatially according to their redox potentials and organized into four complexes (Figure 1). Electrons derived from metabolic reducing equivalents (NADH and FADH_2_) are transferred into the ETC through either complex I or complex II, and eventually pass to molecular oxygen (O_2_) to form H_2_O in complex IV. Electron transport through the mitochondrial ETC is coupled to the transport of protons from the mitochondrial matrix to the mitochondrial intermembrane space, generating an electrochemical proton potential that is utilized by the ATP synthase (complex V) to form ATP (Figure 1). Thermodynamically, all of these electron carriers in their reduced state (standard redox potentials ranging from - 0.320 to + 0.380 V) could pass their electrons to O_2 _(standard redox potential: + 0.815 V) to form superoxide [3]. However, extensive studies with isolated mitochondria and submitochondrial particles detected only a few ROS-forming sites in the mitochondrial ETC (Fig. 1B), namely the ubiquinone site in complex III [4], the N2 iron-sulfur protein [5] or the ubiquinone-binding site [6] in complex I, suggesting that most of the electron carriers in the complexes may be shielded from O_2_. With isolated mitochondria, the complex II substrate succinate supports the highest ROS production rate in the absence of respiratory inhibitors. Most of the succinate-supported ROS production is generated at the flavin mononucleotide (FMN) group in complex I through reversed electron transfer [7-9]. Reversed electron transfer occurs in the absence of ADP when electrons derived from succinate flow in reverse to complex I and reduce NAD^+ ^to NADH. ROS production through reversed electron transfer, which is more likely to occur when the mitochondrial membrane potential is high, is particularly sensitive to inhibition by agents such as ADP and proton ionophore uncouplers which use or dissipate the transmembrane proton gradient. However, the relevance of the ROS-generating sites identified using isolated mitochondria may be different from those producing ROS in living cells is not entirely clear, in part because mitochondria in living cells are simultaneously exposed to a variety of substrates. In addition, many cellular factors that regulate mitochondrial electron transport and ROS production are absent from isolated mitochondria. Therefore, conclusions reached with *in vitro *data may not accurately reflect mitochondrial ROS production in living cells.

In the present report, we examined mitochondrial ROS production in cultured cells under three pathophysiologically relevant situations where mitochondrially generated oxidative stress is directly related to cell death: oxidative glutamate toxicity, state IV respiration (respiration in the absence of ADP) artificially induced with oligomycin, and tumor necrosis factor α (TNFα)-induced cell death. We also tested the effectiveness of various antioxidants on ROS generation and cell death under these situations. It is shown that the mitochondrial sites of ROS generation are stressor-specific and that the accessibility of antioxidants to ROS generated at each site within the ETC is distinct. Based on these results and other evidence in the literature, it is inferred that there are at least four ROS-generating sites in the mitochondrial ETC in living cells: the FMN group of complex I and the three ubiquinone-binding sites in complexes I, II and III.

## Methods

### Materials and Cell Lines

Tissue culture reagents were purchased from Invitrogen (San Diego, CA). 5-(and-6)-chloromethyl-2',7'-dichlorodihydrofluorescein diacetate (CM-H_2_DCFDA), 5,5',6,6'-tetrachloro-1,1',3,3'-tetraethyl-benzimidazolcarbocyanine iodide (JC-1), 4,6'-diamidino-2-phenylindole (DAPI), Hoechst H33342, and propidium iodide were obtained from Molecular Probes (Eugene, OR). Iron 5,10,15,20-tetrakis-4-carboxyphenyl porphyrin (FeTCCP) was from Frontier Scientific (Logan, UT). All other reagents were from Sigma. All drugs were tested at 2-fold serial dilutions higher and lower than that used in the cited publications, and the minimum effective dose was used.

HT-22 cells were derived from the immortalized mouse hippocampal cell line HT-4 [10] and were cultured with DMEM plus 10% fetal bovine serum (FBS). L929 murine fibrosarcoma cells were obtained from the American Type Culture Collection (ATCC, Rockville, MD) and grown in DMEM with 10% FBS. Pancreatin (Invitrogen) was used to dissociate cells from culture dishes.

### Staining Cells with Fluorescent Dyes and Photomicroscopy

To study cellular superoxide level, cells were stained with 5 μM MitoSOX red for 10 min at 37°C and washed three times before imaging. To assess mitochondrial membrane potential, the cells were incubated with 2 μM JC-1 for 30 min at 37°C, and washed three times before imaging. Nuclei were revealed by staining with the cell permeable dye (Hoechst 33342) (10 μM) for 10 min at 37°C. Images were taken with a Leica inverted microscope equipped with a cooled Hamamatsu digital CCD camera (C4742-95) and Openlab imaging software.

To study whether oxidized MitoSOX red by superoxide accumulates in mitochondria, MitoSOX red (5 μM) was oxidized in the absence of cells using superoxide generated from the xanthine/xanthine oxidase system at 37°C for 100 min (100 μM xanthine, 50 mU/ml xanthine oxidase, 2300 U/ml catalase). The reaction was stopped by adding 100 μM allopurinol. HT-22 cells were then incubated in oxidized probe solution for 10 min at 37°C. As a control, a MitoSOX red sample went through the same procedure as above but with the omission of xanthine oxides to prevent the formation of superoxide. Further, extensive information about the superoxide specificity of MitoSOX red can be obtained from the manufacture, Molecular Probes of Invitrogen.

### Cellular ROS Levels

The cellular levels of ROS were determined using CM-H_2_DCFDA and MitoSOX red. A protocol that measures both superoxide and other ROS with CM-H_2_DCFDA and MitoSOX red was developed. The cells were washed once with DMEM and then dissociated from tissue culture dishes with pancretin (Invitrogen, San Diego, CA) in DMEM in the presence of 5 μM CM-H_2_DCFDA and 4 μM MitoSOX red for 10 min at 37°C. After centrifugation, the cell pellets were resuspended and washed once at room temperature phenol red-free, HEPES-buffered DMEM supplemented with 2% dialyzed FBS. The cells were then resuspended in 750 μl of the same buffer containing 1 μg/ml DAPI and kept on ice until flow cytometric analysis. DAPI was used to gate for live cells. Flow cytometric analysis was performed with a Becton-Dickinson LSR three-laser six-color analytic flow cytometer and the data acquisition program CELLQuest™ (Becton-Dickinson, San Jose, CA). Data were collected for 10,000 viable cells after gating out the dead cells using light scattering characteristics and DAPI fluorescence. Median fluorescence intensities (MFI) of the samples were determined. The excitation and emission wavelength used for the dyes are λ_ex_/λ_em _= 345/455 nm for DAPI, λ_ex_/λ_em _= 475/525 nm for dichlorofluorescein (DCF), and λ_ex_/λ_em _= 510/580 nm for MitoSOX red. Color compensation was applied to properly analyze the multicolor data. With the LSR three-laser six-color analytic flow cytometer, the optical layout of this machine dictates that cells pass through and are analyzed first by the 488 nm excitation beam before they encounter the UV beam. Cells typically encounter the UV beam approximately 17 microseconds after they have been analyzed by the 488 nm beam for 10 microseconds, therefore it is physically impossible for the UV beam to affect DCF or MitoSOX fluorescence measured with the 488 nm beam. Photobleaching is therefore minimal and is not a recognized problem in analytical flow cytometers such as the LSR. In all cases controls were done to insure that any added reagents, such as antioxidants, do not directly contribute to the fluorescent signals.

### Determination of Mitochondrial Membrane Potential By Flow Cytometry

JC-1 was used to determine the mitochondrial membrane potential [11]. Cells were washed once with fresh culture medium to remove test compounds before incubating in 2 μM JC-1 for 30 min at 37°C. The cells were then dissociated with pancretin and washed once in room temperature phenol red-free, HEPES-buffered DMEM supplemented with 2% dialyzed FBS. Cell pellets were then resuspended in the same buffer with 1 μg/ml DAPI for the gating of dead cells. Flow cytometric analysis was performed on 10,000 viable cells with a LSR three-laser six-color analytic flow cytometer and CELLQuest™ software (Becton-Dickinson, San Jose, CA). The excitation and emission wavelength used for the dyes are λ_ex_/λ_em _= 345/455 nm for DAPI, λ_ex_/λ_em _= 488 nm/530 nm for the JC-1 monomer and 590 nm for the JC-1 aggregate.

### Cell Viability Assay

For oligomycin- and TNFα-induced cytotoxicity of HT-22 or L929 cells, cell viability was determined by trypan blue (0.4%) exclusion after overnight treatment. About 500 cells were counted for each sample. The LDH release assay was used in some cases according to manufacturer's (Sigma) instructions.

### Statistics

Statistical analysis was performed with GraphPad Prism software (San Diego, CA). Most of the data are presented as mean ± standard error of the mean (SEM) of three independent determinations. In some cases ANOVA analysis was used to evaluate the data followed by a Tukey post-hoc test.

## Results

**Figure 2:**
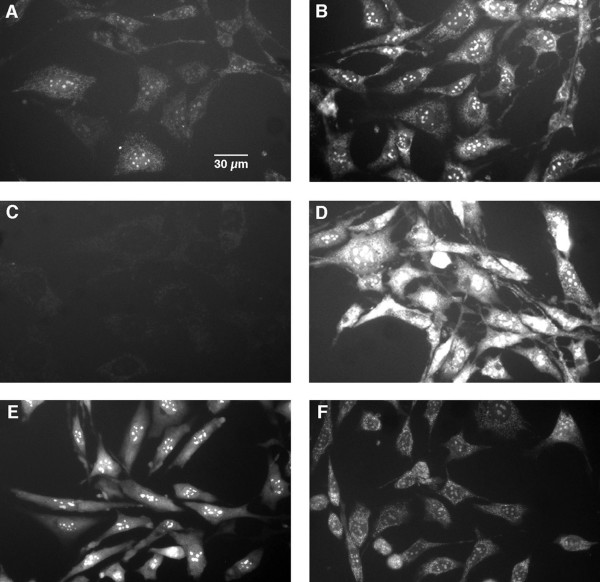
**Assessing Cellular Levels of Superoxide with the Superoxide Indicator MitoSOX Red. HT-22 cells were stained with 5 μM MitoSOX red for 10 min at 37°C before imaging**. *A*: Control. *B*: The cells were treated with 10 μg/ml oligomycin for 2 hr. *C*: The cells were treated with 10 μg/ml oligomycin and 50 μM FeTCPP for 2 hr. *D*: The cells were treated with 1 μM myxothiazol for 2 hr. *E*: The cells were treated with 10 μg/ml oligomycin for 2 hr and stained with MitoSOX red that had been oxidized by superoxide from the xanthine/xanthine oxidase system. *F*: The cells were treated with 10 μg/ml oligomycin for 2 hr and stained with MitoSOX red that went through the same xanthine/xanthine oxidase procedure as in *E *except that xanthine oxidase was omitted to prevent the formation of superoxide.

In order to identify sites of ROS production under conditions of stress, it is first necessary to understand ROS production in normal cells and to verify that the reagents used to study mitochondrial ROS production function in cells as they do with isolated mitochondria. Since respiratory inhibitors have been used extensively to study ROS production by isolated mitochondria, we used these reagents to identify sites of cellular ROS production in the mouse hippocampal nerve cell line HT-22. Cellular ROS were assayed using both 5-(and-6)-chloromethyl-2', 7'-dichlorodihydrofluorescein diacetate (CM-H_2_DCFDA) and MitoSOX red. CM-H_2_DCFDA has been used extensively to measure cellular H_2_O_2 _and other ROS, but it does not measure superoxide directly [12]. MitoSOX red is a selective indicator of mitochondrial superoxide that is better than the commonly used superoxide probe dihydroethidium in terms of both mitochondrial localization and specificity toward superoxide [13]. MitoSOX red is selectively targeted to mitochondria and is able to compete efficiently with superoxide dismutase (SOD) for superoxide. Oxidized MitoSOX red becomes highly fluorescent upon binding nucleic acids, and MitoSOX red preoxidized by superoxide does not accumulate in mitochondria [13]. These characteristics make MitoSOX red a selective indicator of mitochondrial superoxide production. As shown in Figure 2, cellular MitoSOX red staining is limited to mitochondria and 5 to 8 nuclear spots. The fluorescence intensity in both mitochondria and the nuclear spots is greatly increased by the ATP synthase inhibitor oligomycin and the complex III/I inhibitor myxothiazol (Figure 2B and 2D). Iron 5,10,15,20-tetrakis-4-carboxyphenyl porphyrin (FeTCPP), a small molecule SOD mimetic [14], almost completely abolished the oligomycin-induced fluorescence signal, indicating that the fluorescence was indeed caused by superoxide (Figure 2C). The nuclear MitoSOX red fluorescent spots might represent superoxide produced by cytoplasmic/nuclear ROS generator(s) or by superoxide released from mitochondria. The latter possibility is supported by the observation that following oligomycin and myxothiazol treatment, there is an increased intensity of the nuclear spots (Figure 2B and 2D). In confirmation of the findings by Robinson et al. [13]. MitoSOX red pre-oxidized by superoxide from the xanthine/xanthine oxidase system did not accumulate in mitochondria, but showed a diffuse cytoplasmic staining and bright nuclear spots (Figure 2E). In contrast, MitoSOX red that went through the same xanthine/xanthine oxidase treatment except with xanthine oxidase omitted to prevent the formation of superoxide, showed the characteristic mitochondrial staining pattern (Figure 2F). These results confirm that MitoSOX red is selectively taken up by mitochondria and that its fluorescence signal represents mostly mitochondrially-generated superoxide.

**Figure 3:**
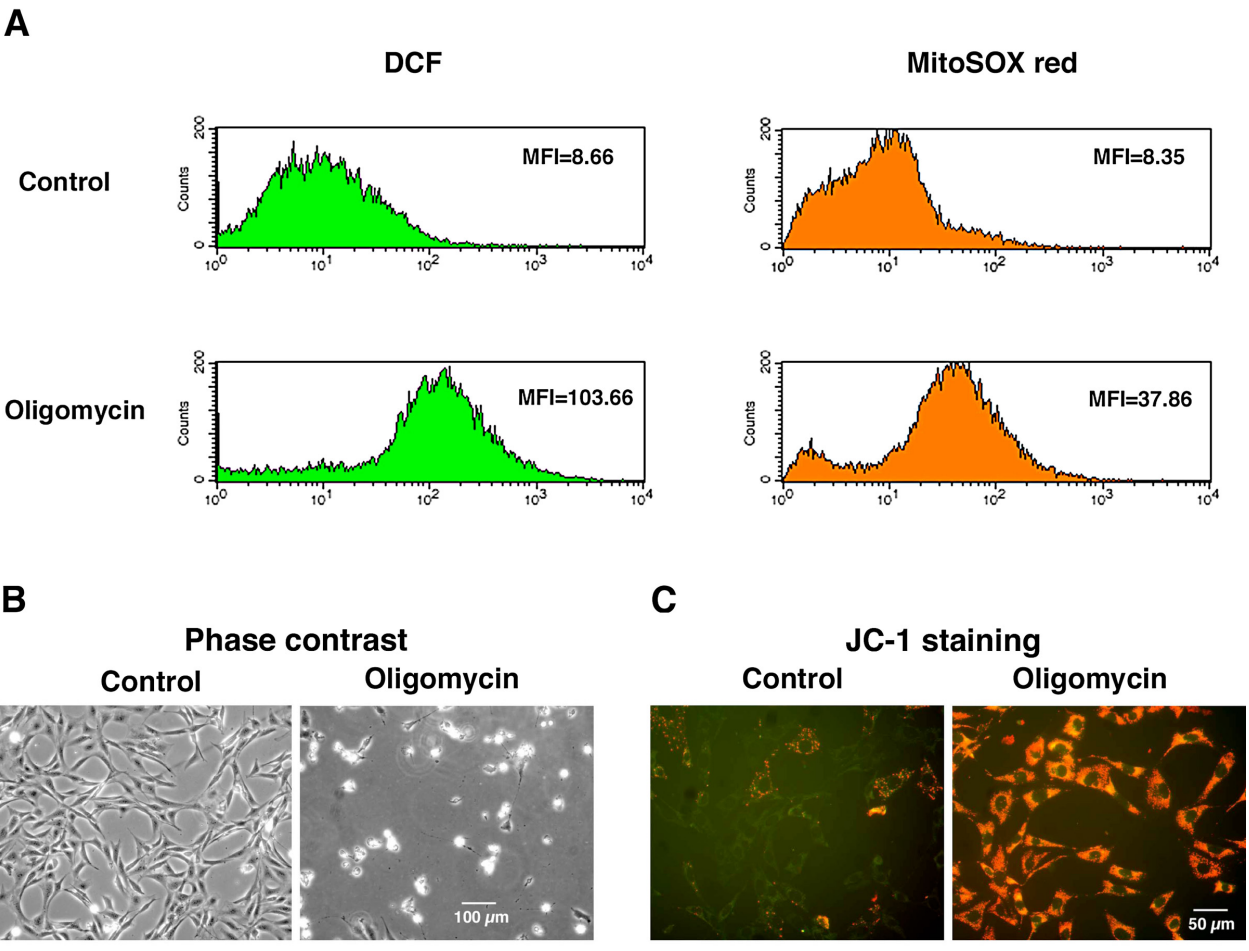
**Oligomycin-induced Cellular ROS Production and Cell Death in HT-22 Cells**. *A*: Flow cytometric analysis of cellular ROS levels with the simultaneous use of CM-H_2_DCFDA and MitoSOX red and the effect of oligomycin treatment (10 μg/ml, 2 hr). DCF: dichlorofluorescein. MFI: median fluorescence intensity. *B*: Phase contrast images. Images were taken after the cells were treated or untreated with 10 μg/ml oligomycin for 8 hr. *C*: JC-1 staining. The cells were treated or untreated with 10 μg/ml oligomycin for 2 hr.

Simultaneous flow cytometric analysis of DCF-detectable ROS and MitoSOX-detectable superoxide revealed a basal level of both cellular superoxide and H_2_O_2 _and other ROS production in HT-22 cells (Figure 3A). Treatment of the cells with various respiratory inhibitors demonstrated the complex nature of ROS production in living cells (Table 1). Consistent with the *in vitro *finding that inhibitors of the quinone-binding site in complex I induce ROS production [6,8], the complex I inhibitors rotenone and piercidin A increased cellular ROS levels by 30 to 120%. Interestingly, amytal, another inhibitor of the quinone-binding site in complex I slightly decreased cellular ROS levels. This may reflect the complex nature of the quinone-binding sites in complex I and the route of electron transfer through these sites, which is not yet completely understood [15]. The complex III inhibitors (antimycin A, myxothiazol and stigmatellin) all increased cellular ROS levels while the inhibition of complex IV with NaN_3 _had no significant effect. These results are consistent with data obtained from isolated mitochondria [6]. The complex II inhibitor theonyltrifluoroacetone (TTFA) increased cellular ROS levels by 30 to 50% and the ATP synthase inhibitor oligomycin increased the cellular ROS levels over 10-fold (Table 1 and Fig. 3A). The proton ionophore carbonyl cynide p-trifluoromethoxyphenylhydrozone (FCCP) and the flavin protein inhibitor diphenyleneiodonium (DPI), both of which inhibit ROS production by isolated mitochondria through reversed electron transfer [12], increased the total cellular ROS levels by 60 to 120%, suggesting that ROS production through reversed electron transfer does not contribute significantly to the basal cellular level in this cellular model. The effect of FCCP and DPI on basal cellular ROS level may be indirect and related to their disturbance of cellular metabolism. The mechanism of this effect is unknown.

**Table 1:** Effects of Respiratory Inhibitors on ROS Levels in HT-22 Cells

Respiratory inhibitors	Site of action	ROS level (% of control)	
		DCF	MitoSOX
Amytal, 100 μM	Complex I	83 ± 10*	92 ± 3
Rotenone, 2 μM	Complex I	129 ± 7*	167 ± 27*
Piercidin A, 1 μM	Complex I	213 ± 40*	221 ± 35*
TTFA, 100 μM	Complex II	146 ± 20*	133 ± 14*
Antimycin A, 2 μM	Complex III	125 ± 10*	123 ± 6*
Stigmatellin, 1 μM	Complex III	111 ± 5	124 ± 6*
Myxothiazol, 1 μM	Complex III and I	172 ± 30*	523 ± 50*
NaN_3_, 5 mM	Complex IV	99 ± 5	95 ± 5
Oligomycin, 10 μg/ml	ATP synthase	904 ± 180*	360 ± 30*
FCCP, 5 μM	H^+ ^ionophore	137 ± 6*	123 ± 6*
DPI, 1 μM	Flavin protein	150 ± 14*	171 ± 18*

HT-22 cells were treated with the respiratory inhibitors at the indicated concentrations for 2 hr at 37°C. Cellular ROS levels were determined with CM-H_2_DCFDA and MitoSOX red as described in the methods section. DCF: dichlorofluorescein. *: Significantly different from control by ANOVA (P < 0.05).

Finally, some inhibitors such as myxothiazol and oligomycin induce differential levels of DCF-detectable ROS and MitoSOX red-detected superoxide. For example, myxothiazol induced a 72% increase in DCF-detectable ROS and a 423% increase in superoxide while oligomycin induced an 800% increase in DCF-detectable ROS and only a 260% increase in superoxide (Table 1). Since MitoSOX red competes efficiently with SOD, these differences may be explained by the relative availability of SOD around the ROS-generating sites affected by these respiratory inhibitors.

The data in Figure 2 and Table 1 confirm that mitochondria can be a major cellular ROS generator in living cells and show that the effects of most respiratory inhibitors on cellular ROS level are consistent with their mitochondrial action as previously revealed with isolated mitochondria. However, the relevance of mitochondrial ROS production induced by respiratory inhibitors to mitochondrial ROS production under various pathophysiological conditions is still not clear. This question was addressed in the following sections.

### Oligomycin-Induced Mitochondrial Oxidative Stress

Studies with isolated mitochondria show that ROS production is the greatest during state IV respiration (respiration in the absence of ADP). State IV respiration reflects conditions where there is an excess of ATP and a deficiency of ADP [9]. The ATP synthase inhibitor oligomycin is often used to fix mitochondria in state IV respiration because the transmembrane proton gradient can no longer be used to convert ADP into ATP, resulting in a highly increased membrane potential. In a pathological condition similar to oligomycin treatment, the T8993G mutation in the mitochondrial ATPase-6 gene inhibits ATP synthase activity and causes high mitochondrial membrane potential, ROS over-production and neurogenic ataxia retinitis pigmentosa [16].

Oligomycin induced the highest cellular ROS levels (over 10-fold) of all the respiration inhibitors tested (Table 1) and is also cytotoxic (Figure 3B). As expected, oligomycin increases the mitochondrial membrane potential as indicated by JC-1 staining (Figure 3C). The site of oligomycin-induced ROS generation was investigated using additional respiratory inhibitors. As shown in Table 2, the only respiratory inhibitor that significantly reduced oligomycin-induced ROS production is the complex II inhibitor theonyltrifluoroacetone (TTFA), which interferes with the quinone-binding site of complex II. TTFA also partially protects the cells from oligomycin-induced cell death (Table 2). This result suggests that about 20% of the ROS induced by oligomycin comes from the ubiquinone-binding site of complex II. The proton ionophore uncoupler FCCP and the flavin protein inhibitor DPI do not have a significant effect on oligomycin-induced ROS production and cell death, suggesting that ROS production at the FMN group of complex I through reversed electron transfer does not contribute significantly to oligomycin-induced ROS production and cytotoxicity. It is not clear at present where the rest of the oligomycin-induced ROS is produced, but, as described in the Discussion, it is likely from the quinone-binding sites in complex I and/or complex III.

**Table 2:** Effects of Respiratory Inhibitors and Antioxidants on Oligomycin-Induced Cell Death and ROS Production

Treatments	Cell Viability (% of Control)		Percent of Oligomycin-Induced ROS Level	
	Trypan	LDH	DCF	MitoSOX
Oligomycin (O), 10 μg/ml	0	0	100	100
O + Amytal, 100 μM	0	0	97 ± 3	93 ± 4
O + Rotenone, 2 μM	0	0	90 ± 5	97 ± 3
O + TTFA, 100 μM	41 ± 9*	52 ± 9*	84 ± 6*	79 ± 6*
O + Antimycin A, 2 μM	0	0	92 ± 5	114 ± 10
O + Stigmatellin, 1 μM	0	0	96 ± 5	107 ± 8
O + FCCP, 5 μM	0	0	90 ± 6	106 ± 6
O + DPI, 1 μM	0	0	97 ± 5	132 ± 8*
O + Vitamin E, 100 μM	93 ± 4*	100*	60 ± 5*	59 ± 6*
O + 2,2,5,7,8-Pentamethyl-	0	0	98 ± 5	97 ± 3
6-chromanol, 100 μM				
O + Vitamin C, 100 μM	0	0	85 ± 7	91 ± 7

Cellular ROS levels were determined in HT-22 cells with CM-H_2_DCFDA and MitoSOX red after 2 hr of treatment (Figure 3A) and inhibitors were added at the same time as oligomycin. Cell viability was determined after overnight treatment either by the trypan blue assay or LDH release assay. *: Significantly different from control by ANOVA (P < 0.05).

Oligomycin-induced cell death is blocked by the hydrophobic antioxidant vitamin E. However, a variety of relatively hydrophilic antioxidants including vitamin C, some flavonoids (e.g. kaempferol, luteolin, quercetin and baicalein), N-acetylcysteine, butylated hydroxyanisole (BHA), butylated hydroxytoluene (BHT) and nordihydroguaiaretic acid did not block oligomycin-induced cell death at concentrations between 10 and 100 μM (Table 2 and Table 3), suggesting that most of the cytotoxic ROS is released into the hydrophobic domain of the mitochondrial membrane that is not accessible to hydrophilic antioxidants. Indeed, as shown in Table 2, vitamin E markedly inhibited oligomycin-induced ROS production while the hydrophilic vitamin E analog 2,2,5,7,8-pentamethyl-6-chromanol had no significant effect. The superoxide scavenger FeTCPP at 50 μM almost completely blocked oligomycin cytotoxicity (Table 3), suggesting that superoxide may be the damaging ROS. Vitamin E is not known to be an efficient superoxide scavenger. It is therefore possible that the inhibition of oligomycin-induced superoxide by vitamin E may be more related to an ability to inhibit superoxide production than its ability to scavenge superoxide.

**Table 3:** The Effects of Commonly Used Antioxidants or Inhibitors of ROS-Generating Enzymes on TNFα-, Glutamate- and Oligomycin-Induced Cytotoxicity.

	Cell Viability (% of control)		
Antioxidants or inhibitors	TNFα	Glutamate	Oligomycin
No antioxidant or inhibitor	0	0	0
Vitamin E, 100 μM	0	100*	93 ± 4*
Vitamin C, 100 μM	0	100*	0
BHA, 100 μM	95 ± 3*	92 ± 4*	0
BHT, 100 μM	0	45 ± 6*	0
Idebenone, 10 μM	0	97 ± 3*	0
N-Acetylcysteine, 100 μM	0	100*	0
Quercetin, 10 μM	0	96 ± 4*	0
L-NAME^a^, 100 μM	0	0	0
Allopurinol, 100 μM	0	0	12 ± 3*
Desferoxamine, 100 μM	0	91 ± 4*	0
FeTCPP, 50 μM	0	0	95 ± 4*

TNFα cytotoxicity was assayed with L929 cells as described in the legend of Figure 3. Oxidative glutamate toxicity (5 mM) and oligomycin (10 μg/ml)-induced cytotoxicity were determined with HT-22 cells after overnight treatment. ^a^L-NAME: N^G^-monomethyl-L-arginine methyl ester.

*: Significantly different from "No antioxidant or inhibitor" by ANOVA (P < 0.05).

### Oxidative Glutamate Toxicity Induces Dramatic ROS Production From the FMN Group of Complex I

A reduction in the level of intracellular glutathione (GSH) is associated with neurodegenerative diseases such as Parkinson's disease [17]. A robust model of mitochondrial ROS-induced cell death caused by GSH depletion is oxidative glutamate toxicity. Oxidative glutamate toxicity has been extensively studied using the mouse hippocampal cell line HT-22 and immature primary cortical neurons [18,19]. In this model, extracellularly added glutamate inhibits cystine uptake through the cystine/glutamate antiporter, resulting in the depletion of intracellular cysteine and GSH. When the cellular GSH level drops below 20% of the control, an explosive generation of ROS occurs that is required for the subsequent cell death. The source of this robust ROS generation is from mitochondria because both the ROS production and the subsequent cell death are blocked by the proton ionophore FCCP that dissipates the mitochondrial transmembrane proton gradient [19]. These findings suggest that ROS production in oxidative glutamate toxicity might proceed through reversed electron transfer at the FMN group of complex I as with isolated mitochondria [8]. If this is the case, then the mitochondrial membrane potential should be increased following glutamate treatment and the flavin protein inhibitor DPI should be able to inhibit both ROS production and the subsequent cell death (Figure. 1).

**Figure 4:**
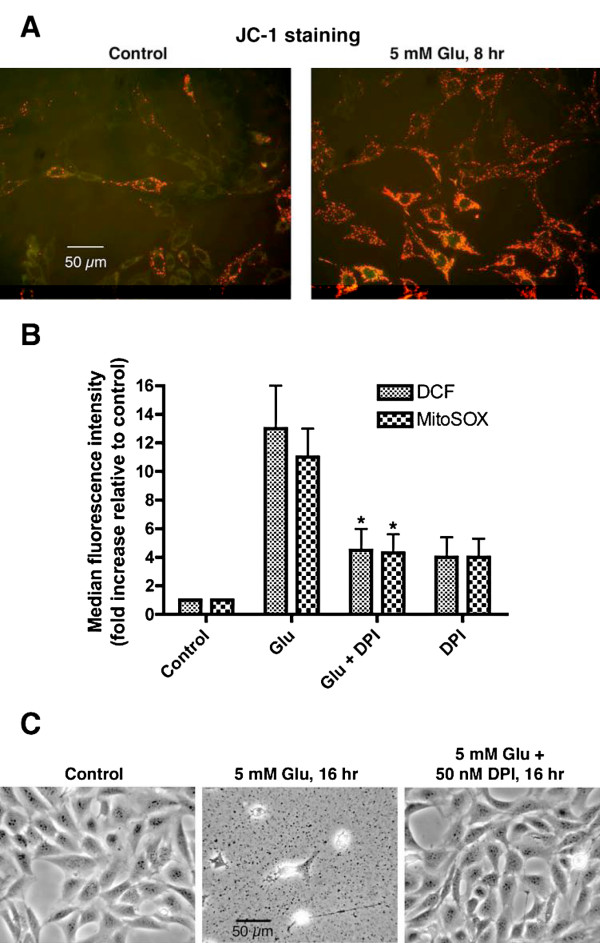
**Oxidative Glutamate (Glu) Toxicity Increases Mitochondrial Membrane Potential and Cellular ROS Levels: Inhibition by Diphenyleneiodonium (DPI)**. *A*: HT-22 cells were treated as indicated, followed by JC-1 staining to reveal the mitochondrial membrane potential. *B*: HT-22 cells were treated for 8 hr with 5 mM glutamate or 5 mM glutamate plus 50 nM DPI. Cellular ROS levels were then measured with flow cytometry using CM-H_2_DCFDA and MitoSOX red. *: Significantly different from glutamate-treated cells by paired Student's t-test (p < 0.05). *C*: Phase contrast images of HT-22 cells treated with glutamate or glutamate plus DPI.

As shown in Figure 4, the addition of glutamate to HT-22 cells results in hyperpolarization of the mitochondria membrane (Figure 4A) and an over 20-fold increase in total cellular ROS levels as measured by the fluorescent ROS probes CM-H_2_DCFDA and MitoSOX red (Figure 4B). The flavin protein inhibitor DPI not only prevents glutamate-induced ROS production but also the subsequent cell death (Figure 4B and 4C). Despite the fact that complex II also contains a flavin group, low concentrations of DPI selectively inhibit the activity of complex I in isolated mitochondria [8] in intact cells [20] and in animals [21]. It is possible that DPI at the concentration used (50 nM) also inhibited other ROS-generating flavin oxidases such as nitric oxide synthase, xanthine oxidase and monoamine oxidase. However, specific inhibitors of these flavin oxidases at effective concentrations do not inhibit the ROS production and the cell death in oxidative glutamate toxicity (Table 3 and data not shown) [also see [19]]. Because the proton ionophore FCCP blocks ROS production and cell death in oxidative glutamate toxicity [19] and FCCP is also known to block the ROS production by isolated mitochondria through reversed electron transfer at the FMN group of complex I [8] it follows from the results in Figure 4 that the FMN group of complex I is the major source of ROS production in oxidative glutamate toxicity.

### TNFα Induces ROS Production From the Quinone-Binding Sites of Complexes I and II

**Figure 5:**
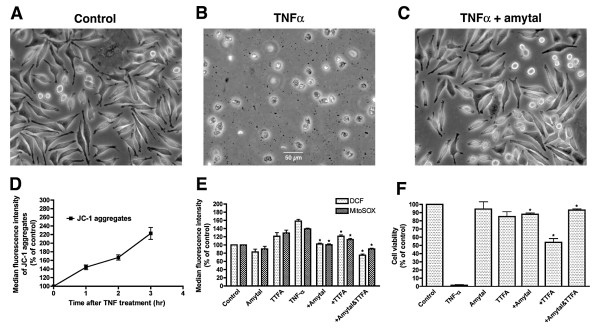
**Effects of TNFα on Mitochondrial Membrane Potential, Cellular ROS Levels and Cell Viability in L929 Cells**. *A*: Control. *B*: The cells were treated with 10 ng/ml TNFα and 1 μg/ml actinomycin D for 16 hr. *C*: The cells were treated with 10 ng/ml TNFα and 1 μg/ml actinomycin D plus 100 μM amytal for 16 hr. *D*: The cells were treated with 10 ng/ml TNFα and 1 μg/ml actinomycin D for the indicated periods of time. *E*: The cells were treated with 10 ng/ml TNFα and 1 μg/ml actinomycin D alone or plus 100 μM of the indicated agents for 3 hr. *F*: The cells were treated with 10 ng/ml TNFα and 1 μg/ml actinomycin D alone or plus 100 μM of the indicated agents for 16 hr. *: Significantly different from TNFα-treated cells by paired Student's t-test (p < 0.05).

TNFα is a major inflammatory cytokine that induces, among many other activities, the death of sensitive nerve cells [see, for example, McCoy, 2006] [22]. TNFα causes necrotic cell death in mouse L929 fibrosarcoma cells through an increase in mitochondrial ROS generation as detected by the ROS probe dihydrorhodamine 123 and a variety of other methods [23]. The L929 cells are used here instead of nerve because of the extensive literature on TNFα toxicity with these cells. It has been suggested that TNFα-induced mitochondrial ROS is produced from the ubiquinone-binding sites of both complex I and complex II because the complex I inhibitor amytal and the complex II inhibitor TTFA block TNFα-induced cell death and because both amytal and TTFA are known to act at the ubiquinone-binding sites [23]. We used the TNFα/L929 cell system (Figure 5A and 5B) to further investigate the sites of TNFα-induced mitochondrial ROS generation by the simultaneous detection of cellular DCF-detectable ROS and MitoSOX-detectable superoxide. As shown in Figure 5D and 5E, TNFα treatment induced a time dependent increase in both mitochondrial membrane potential as defined by JC-1 fluorescence and an ~100% increase in cellular ROS levels after 3 hr of treatment. The complex I inhibitor amytal and the complex II inhibitor TTFA reduced both TNFα-induced ROS increase and cell death, and the combination of amytal and TTFA completely blocked TNFα-induced ROS and cell death (Figure 5C, and 5F). Other respiratory inhibitors tested, including rotenone, piercidin A, antimycin A, stigmatellin, myxothiazol, NaN_3_, FCCP and DPI, had no significant effects on TNFα-induced ROS increase or cell death. These results are consistent with the suggestion that TNFα induces mitochondrial ROS production from the ubiquinone-binding sites of both complex I and complex II [22]. TNFα-induced cell death in L929 cells is blocked by the antioxidant BHA (Table 3), but is not inhibited by a variety of both hydrophobic and hydrophilic antioxidants including vitamin E, vitamin C, flavonoids (kaempferol, luteolin, quercetin and baicalein), N-acetylcysteine, BHT and nordihydroguaiaretic acid. Therefore, TNFα-induced ROS is released into a domain that is not accessible to these antioxidants.

## Discussion

The work presented above analyzes mitochondrial ROS generation in cells under both normal conditions and under three situations reflecting nerve cell death where mitochondrially generated oxidative stress is directly involved: TNFα-induced cell death, oxidative glutamate toxicity and oligomycin-induced state IV respiration. These experiments revealed the complex nature of mitochondrial ROS production in cells. The three different stress-induced situations described in this study all resulted in an increase of mitochondrial membrane potential, yet the sites of ROS production and the amounts of ROS produced are all very different. Based on in vitro studies with isolated mitochondria and respiratory inhibitors, it has been estimated that between 0.15% to 4% of the oxygen consumed by mitochondria is converted to superoxide [24]. However, the amount of oxygen that is converted to superoxide by mitochondria *in vivo *is determined by both the respiratory state and extrinsic factors. A variety of factors and conditions are known to modulate mitochondrial ROS production, including inflammatory cytokines such as TNF-α and IL-1 [25] mitochondrial thiol status [26], oncogenes [27], lipid mediators such as 15-deoxy-Δ^12,14^-prostaglandin J2 [28], excitotoxicity [29] and toxic chemicals such as dioxin [30]. It is therefore important to understand how these various stresses modulate mitochondrial ROS production using living cells and *in vivo *model systems.

The major difficulty in studying mitochondrial ROS production in intact cells has been in being certain that cellular ROS detected with a fluorescence probe is from mitochondria and not from other ROS generators. This is because there have been no selective indicators for mitochondrial ROS in intact cells and because some of the commonly used respiratory inhibitors may have targets other than mitochondria in intact cells. However, the newly developed MitoSOX red used in this study is a good selective indicator of mitochondrial superoxide [13], and the non-mitochondrial actions of the respiratory inhibitors could be examined to determine if their effects are consistent with their intended targets.

TTFA is commonly assumed to act upon the quinone-binding sites of complex II, but it also inhibits malate dehydrogenase [31] and liver/plasma carboxylesterase [32] at the concentrations used here (50 to 100 μM). Inhibition of carboxylesterase could limit the availability of DCF to react with ROS and lead to lowered estimate of DCF-detectable ROS. However, in our study, cellular ROS is measured with both CM-H2DCFDA and MitoSOX red, and MitoSOX red is not an ester and therefore not sensitive to carboxylesterase inhibition. Since no differential effects of TTFA on DCF and MitoSOX red signals are detected (Table 1 and 2, Figure 5), the effect of TTFA on carboxylesterase does not appear to be a concern with the cellular models used here. Other inhibitors of malate dehydrogenase (thyroxine and chlorothricin) also failed to mimic the effects of TTFA on oligomycin- and TNFα-induced ROS production and cell death (data not shown). These results suggest that the effect of TTFA on cellular ROS production is consistent with its action on the quinone-binding sites of complex II. Similarly, the effect of the flavin protein inhibitor DPI on oxidative glutamate toxicity is most consistent with its selective interaction with the FMN group of complex I.

The oxidative glutamate toxicity data show that the FMN group in complex I can be a robust ROS-generating site in living cells under conditions that favor reversed electron transfer such as low intracellular cysteine/GSH. The studies on oligomycin- and TNFα-induced ROS production indicate that the ubiquinone-binding sites in both complex I and complex II can also serve as ROS-generating sites in intact cells [23]. Although lacking a pathophysiological example, it is likely that the ubiquinone-binding site(s) in complex III is also a ROS-generating site in living cells. Therefore, there are at least three and possibly four ROS-generating sites in the mitochondrial ETC that can produce ROS in cells, namely, the FMN group of complex I and the three ubiquinone-binding sites in complexes I, II and III (Figure 1). However, there are at least two quinone-binding sites within each complex and the route of electron transfer through these sites is not completely understood [15]. The details of ROS generation around these quinone-binding sites as well as their modulation remain to be established.

Although most of the electron carriers in the mitochondrial ETC have the potential to pass electrons to O_2 _to form superoxide, the fact that only a few actually generate superoxide suggests that most are shielded from O_2_. Because both FMN and ubiquinone have access to O_2 _and both can form a stable semiquinone free radical state that can pass electrons to O_2_, it is probably not a coincidence that they have been associated with ROS production. However, whether ubisemiquinone itself or the iron-sulfur center at the quinone-binding site is the source of superoxide is still not resolved [5].

Our data suggest that the accessibility of the four mitochondrial ETC ROS-generating sites to commonly used antioxidants as well as an antioxidant's ability to block toxicity is more specific than commonly assumed. While ROS produced around the FMN group in complex I can be scavenged by a variety of both hydrophilic and hydrophobic antioxidants that also block cell death in oxidative glutamate toxicity (Table 3) (see also [19]), ROS generated around the quinone-binding sites in complexes I and II, as is seen following TNFα and oligomycin treatment, are not accessible to most of the commonly used antioxidants shown in Table 3. Therefore specific antioxidants with accessibility to ROS generated at the quinone-binding sites or specific inhibitors of ROS production at these sites are needed to reduce ROS production in these pathophysiological situations.

ROS production around the quinone-binding sites in complex I has long been recognized with studies using isolated mitochondria [5] and is believed to be important in the pathogenesis of Parkinson's disease and a number of other diseases associated with complex I deficiency [33]. Most complex I inhibitors acting around the quinone-binding sites of complex I (such as rotenone and piercidin A) increase ROS production by themselves (Table 1). However, the clinically useful amytal is an interesting exception. It both decreased cellular ROS level in unstressed cells (Table 1) and inhibited ROS production by TNFα (Figure 5E). These results suggest that it may be possible to find additional, more potent compounds that inhibit ROS production at the quinone-binding sites of complex I.

Studies with isolated mitochondria have not yet identified complex II as a ROS-generating site, but medical evidence suggests that complex II can be an important source of pathological ROS production. Genetic defects in human complex II result in a number of diseases that often manifest as tumors and neurological disorders associated with the production of ROS (for review, see [34]). Ishii et al. [35] also found that a mutation in the cytochrome b of complex II causes oxidative stress and rapid aging in nematodes. More importantly, mitochondrial ROS overproduction resulting from diabetic hyperglycemia is largely blocked by the complex II inhibitor TTFA [36]. Therefore, ROS production by complex II is not only important in rare mitochondrial diseases but also important in a major disease such as type II diabetes. In the present study, we found that TTFA inhibited the cellular ROS production induced by both oligomycin and TNFα, further supporting the idea that complex II can be an important source of ROS in cells. Sun et al. [37] have recently reported the first crystal structure of mammalian complex II and demonstrated two quinone-binding sites through complex II-TTFA complex. This information should be very useful for understanding ROS production by complex II and for designing inhibitors of complex II ROS production.

A variety of other diseases are also associated with mitochondrial respiratory chain deficiencies [2]. Using elevated expression of mitochondrial manganese SOD as a marker of increased superoxide production, skin fibroblasts or muscle biopsies from patients with mitochondrial respiratory chain deficiencies in complexes I, II or V were found to have increased superoxide production [16]. Surprisingly, a literature search failed to find reports about superoxide overproduction caused by complex III mutations even though complex III is often suggested to be the largest source of mitochondrial ROS based on studies using isolated mitochondria and the complex III inhibitor antimycin A. In contrast, complex V deficiency caused by the T8993G mutation in the mitochondrial ATPase-6 gene induces ROS overproduction and neurogenic ataxia retinitis pigmentosa [16]. Oligomycin treatment mimics this pathological condition. We have found that oligomycin-induced ROS production and cell death is blocked by vitamin E, suggesting that vitamin E treatment may be beneficial to patients with complex V deficiency.

## Conclusion

Our study has demonstrated the complex nature of mitochondrial ROS production in cells under several pathophysiological conditions. It is inferred that there are at least four ROS-generating sites in the mitochondrial ETC that can produce ROS in cells: the FMN group of complex I and the three ubiquinone-binding sites in complex I, II and III. The accessibility of commonly used antioxidants to these ROS-generating sites varies tremendously. Since ROS production around the ubiquinone-binding sites within complex I to III is associated with major diseases and is not accessible to existing antioxidants, new drugs that can inhibit ROS production from these sites may prove to be the most useful in treating mitochondrial ROS-related diseases such as Parkinson's disease and type II diabetes.

## Abbreviations

BHA: butylated hydroxyanisole'; BHT: butylated hydroxytoluene (BHT); CM-H_2_DCFDA: dichlorodihydrofluorescein diacetate; DAPI: diamidino-2-phenylindole; DCF: dichlorofluorocine; DMEM: Dulbecco's modified Eagle's medium; DPI: diphenyleneiodonium; ETC: electron transport chain; FBS: fetal bovine serum; FCCP: proton ionophore carbonyl cynide p-trifluoromethoxyphenylhydrozone; FeTCCP: Iron 5,10,15,20-tetrakis-4-carboxyphenyl porphyrin; FeTCPP: iron(III)-tetrakis(*p*-carboxyphenyl)porphyrin; FMN: flavin mononucleotide; GLU: Glutamate; GSH: glutathione; HEPES: 4-(2-hydroxyethyl)-1-piperazine ethanesulfonic acid); JC-1: tetrachloro-1,1',3,3'-tetraethyl-benzimidazolcarbocyanine iodide; MFI: median fluorescence intensity; ROS: Reactive oxygen species; SEM: standard error of the mean; SOD: superoxide dismutase; TNFα: tumor necrosis factor α; TTFA: theonyltrifluoroacetone; UV: ultraviolet.

## Competing interests

The authors, Yuanbin Liu and David R. Schubert, declare that no competing interests exist.

## Authors' contributions

YL did most of the experimental work, while DS did some of the cell death assays, participated in the design of the study, and helped write the manuscript.

## References

[B1] Schriner SE Linford NJ Martin GM Treuting P Ogburn CE Emond M Coskun PE Ladiges W Wolf N Van Remmen H Wallace DC Rabinovitch PS Extension of murine life span by overexpression of catalase targeted to mitochondria Science 2005 308 1909 1911 10.1126/science.1106653 15879174

[B2] Wallace DC A mitochondrial paradigm of metabolic and degenerative diseases, aging, and cancer: A dawn for evolutionary medicine Annu Rev Genet 2005 39 359 407 10.1146/annurev.genet.39.110304.095751 PMC2821041 16285865

[B3] Wilson DF Erecinska M Dutton PL Thermodynamic relationships in mitochondrial oxidative phosphorylation Annu Rev Biophys Bioeng 1974 3 203 230 10.1146/annurev.bb.03.060174.001223 4153883

[B4] Turrens JF Alexandre A Lehninger AL Ubisemiquinone is the electron donor for superoxide formation by complex iii of heart mitochondria Arch Biochem Biophys 1985 237 408 414 10.1016/0003-9861(85)90293-0 2983613

[B5] Genova ML Ventura B Giuliano G Bovina C Formiggini G Parenti Castelli G Lenaz G The site of production of superoxide radical in mitochondrial complex i is not a bound ubisemiquinone but presumably iron-sulfur cluster n2 FEBS Lett 2001 505 364 368 10.1016/s0014-5793(01)02850-2 11576529

[B6] Lambert AJ Brand MD Inhibitors of the quinone-binding site allow rapid superoxide production from mitochondrial nadh:Ubiquinone oxidoreductase (complex i) J Biol Chem 2004 279 39414 39420 10.1074/jbc.M406576200 15262965

[B7] Cino M Del Maestro RF Generation of hydrogen peroxide by brain mitochondria: The effect of reoxygenation following postdecapitative ischemia Arch Biochem Biophys 1989 269 623 638 10.1016/0003-9861(89)90148-3 2919886

[B8] Liu Y Fiskum G Schubert D Generation of reactive oxygen species by the mitochondrial electron transport chain J Neurochem 2002 80 780 787 10.1046/j.0022-3042.2002.00744.x 11948241

[B9] Kudin AP Bimpong-Buta NY Vielhaber S Elger CE Characterization of superoxide-producing sites in isolated brain mitochondria J Biol Chem 2004 279 4127 4135 10.1074/jbc.M310341200 14625276

[B10] Morimoto BH Koshland DE Jr Induction and expression of long- and short-term neurosecretory potentiation in a neural cell line Neuron 1990 5 875 880 10.1016/0896-6273(90)90347-i 1980069

[B11] Reers M Smiley ST Mottola-Hartshorn C Chen A Lin M Chen LB Mitochondrial membrane potential monitored by jc-1 dye Methods Enzymol 1995 260 406 417 10.1016/0076-6879(95)60154-6 8592463

[B12] LeBel CP Ischiropoulos H Bondy SC Evaluation of the probe 2',7'-dichlorofluorescin as an indicator of reactive oxygen species formation and oxidative stress Chem Res Toxicol 1992 5 227 231 10.1021/tx00026a012 1322737

[B13] Robinson KM Janes MS Beckman JS The selective detection of mitochondrial superoxide by live cell imaging Nat Protoc 2008 3 941 947 10.1038/nprot.2008.56 18536642

[B14] Patel M Day BJ Metalloporphyrin class of therapeutic catalytic antioxidants Trends Pharmacol Sci 1999 20 359 364 10.1016/s0165-6147(99)01336-x 10462758

[B15] Ohnishi T Iron-sulfur clusters/semiquinones in complex i Biochim Biophys Acta 1998 1364 186 206 10.1016/s0005-2728(98)00027-9 9593887

[B16] Geromel V Kadhom N Cebalos-Picot I Ouari O Polidori A Munnich A Rotig A Rustin P Superoxide-induced massive apoptosis in cultured skin fibroblasts harboring the neurogenic ataxia retinitis pigmentosa (narp) mutation in the atpase-6 gene of the mitochondrial DNA Hum Mol Genet 2001 10 1221 1228 10.1093/hmg/10.11.1221 11371515

[B17] Sian J Dexter DT Lees AJ Daniel S Agid Y Javoy-Agid F Jenner P Marsden CD Alterations in glutathione levels in parkinson's disease and other neurodegenerative disorders affecting basal ganglia Ann Neurol 1994 36 348 355 10.1002/ana.410360305 8080242

[B18] Murphy TH Baraban JM Glutamate toxicity in immature cortical neurons precedes development of glutamate receptor currents Brain Res 1990 57 146 150 10.1016/0165-3806(90)90195-5 1982523

[B19] Tan S Sagara Y Liu Y Maher P Schubert D The regulation of reactive oxygen species production during programmed cell death J Cell Biol 1998 141 1423 1432 10.1083/jcb.141.6.1423 PMC2132785 9628898

[B20] Li Y Trush MA Diphenyleneiodonium, an nad(p)h oxidase inhibitor, also potently inhibits mitochondrial reactive oxygen species production Biochem Biophys Res Commun 1998 253 295 299 10.1006/bbrc.1998.9729 9878531

[B21] Holland PC Clark MG Bloxham DP Lardy HA Mechanism of action of the hypoglycemic agent diphenyleneiodonium J Biol Chem 1973 248 6050 6056 4726296

[B22] McCoy MK Martinez TN Ruhn KA Szymkowski DE Smith CG Botterman BR Tansey KE Tansey MG Blocking soluble tumor necrosis factor signaling with dominant-negative tumor necrosis factor inhibitor attenuates loss of dopaminergic neurons in models of parkinson's disease J Neurosci 2006 26 9365 9375 10.1523/JNEUROSCI.1504-06.2006 PMC3707118 16971520

[B23] Schulze-Osthoff K Bakker AC Vanhaesebroeck B Beyaert R Jacob WA Fiers W Cytotoxic activity of tumor necrosis factor is mediated by early damage of mitochondrial functions. Evidence for the involvement of mitochondrial radical generation J Biol Chem 1992 267 5317 5323 1312087

[B24] St-Pierre J Buckingham JA Roebuck SJ Brand MD Topology of superoxide production from different sites in the mitochondrial electron transport chain J Biol Chem 2002 277 44784 44790 10.1074/jbc.M207217200 12237311

[B25] Wong GH Elwell JH Oberley LW Goeddel DV Manganous superoxide dismutase is essential for cellular resistance to cytotoxicity of tumor necrosis factor Cell 1989 58 923 931 10.1016/0092-8674(89)90944-6 2476237

[B26] Taylor ER Hurrell F Shannon RJ Lin TK Hirst J Murphy MP Reversible glutathionylation of complex i increases mitochondrial superoxide formation J Biol Chem 2003 278 19603 19610 10.1074/jbc.M209359200 12649289

[B27] Lee AC Fenster BE Ito H Takeda K Bae NS Hirai T Yu ZX Ferrans VJ Howard BH Finkel T Ras proteins induce senescence by altering the intracellular levels of reactive oxygen species J Biol Chem 1999 274 7936 7940 10.1074/jbc.274.12.7936 10075689

[B28] Martinez B Perez-Castillo A Santos A The mitochondrial respiratory complex i is a target for 15-deoxy-delta12,14-prostaglandin j2 action J Lipid Res 2005 46 736 743 10.1194/jlr.M400392-JLR200 15654126

[B29] Reynolds IJ Hastings TG Glutamate induces the production of reactive oxygen species in cultured forebrain neurons following nmda receptor activation Journal of Neuroscience 1995 15 3318 3327 10.1523/JNEUROSCI.15-05-03318.1995 PMC6578215 7751912

[B30] Shen D Dalton TP Nebert DW Shertzer HG Glutathione redox state regulates mitochondrial reactive oxygen production J Biol Chem 2005 280 25305 25312 10.1074/jbc.M500095200 15883162

[B31] Gunter TE Pfeiffer DR Mechanisms by which mitochondria transport calcium Am J Physiol 1990 258 C755 786 10.1152/ajpcell.1990.258.5.C755 2185657

[B32] Zhang JG Fariss MW Thenoyltrifluoroacetone, a potent inhibitor of carboxylesterase activity Biochem Pharmacol 2002 63 751 754 10.1016/s0006-2952(01)00871-1 11992644

[B33] Pitkanen S Robinson BH Mitochondrial complex i deficiency leads to increased production of superoxide radicals and induction of superoxide dismutase J Clin Invest 1996 98 345 351 10.1172/JCI118798 PMC507436 8755643

[B34] Rustin P Rotig A Inborn errors of complex ii--unusual human mitochondrial diseases Biochim Biophys Acta 2002 1553 117 122 10.1016/s0005-2728(01)00228-6 11803021

[B35] Ishii N Fujii M Hartman PS Tsuda M Yasuda K Senoo-Matsuda N Yanase S Ayusawa D Suzuki K A mutation in succinate dehydrogenase cytochrome b causes oxidative stress and ageing in nematodes Nature 1998 394 694 697 10.1038/29331 9716135

[B36] Nishikawa T Edelstein D Du XL Yamagishi S Matsumura T Kaneda Y Yorek MA Beebe D Oates PJ Hammes HP Giardino I Brownlee M Normalizing mitochondrial superoxide production blocks three pathways of hyperglycaemic damage Nature 2000 404 787 790 10.1038/35008121 10783895

[B37] Sun F Huo X Zhai Y Wang A Xu J Su D Bartlam M Rao Z Crystal structure of mitochondrial respiratory membrane protein complex ii Cell 2005 121 1043 1057 10.1016/j.cell.2005.05.025 15989954

